# Alterations in cognitive performance during passive hyperthermia are task dependent

**DOI:** 10.3109/02656736.2010.516305

**Published:** 2011-01-21

**Authors:** Nadia Gaoua, Sebastien Racinais, Justin Grantham, Farid El Massioui

**Affiliations:** 1 Research and Education Centre, ASPETAR, Qatar Orthopaedic and Sports Medicine Hospital, Doha, Qatar; 2 Laboratoire de Psychologie et de Neurosciences Groupe IME, Paris, France; 3 Laboratoire Cognition Humaine et Artificielle, UFR de Psychologie, Université Paris 8, France

**Keywords:** cognitive capacity, heat stress, task type, temperature

## Abstract

The objectives of this study were to (1) assess the effect of passive heating upon attention and memory task performance, and (2) evaluate the effectiveness of the application of cold packs to the head on preserving these functions. Using a counterbalance design 16 subjects underwent three trials: a control (CON, 20°C, 40% rH), hot (HOT, 50°C, 50% rH) and hot with the head kept cool (HHC). In each condition, three attention tests and two memory tests were performed. Mean core, forehead and tympanic temperatures were all significantly higher (*p*< 0.05) during HOT (38.6° ±0.1°, 39.6° ±0.2° and 38.8°±0.1°C, respectively) and HHC (38°±0.2, 37.7°±0.3° and 37.7°C, respectively) than in CON (37.1°±0.6°, 33.3° ±0.2° and 35.9°±0.3°C, respectively). Results indicate that there was impairment in working memory with heat exposure (*p* < 0.05) without alteration in attentional processes. The regular application of cold packs only prevented the detrimental effect of hyperthermia on short-term memory. Our results show that impairments in cognitive function with passive hyperthermia and the beneficial effect of head cooling are task dependent and suggests that exposure to a hot environment is a competing variable to the cognitive processes.

## Introduction

Hyperthermia can result from extreme uncompen-sated heat exposure. However, methodological discrepancies between studies have made it difficult to conclude whether hyperthermia does [1-5] or does not [6-8] adversely affect cognitive function. Such inconsistencies include; the severity and duration of temperature exposure, the methodology used to induce hyperthermia, the complexity and duration of the cognitive tasks and the skill level of the participants [9-11]. The following examples highlight some of the discrepancies between studies. Military exercises that induced a maximum rise in core temperature to 38.2°C did not affect reaction time and response accuracy in a number and name checking task [8]. Decision-making can be adversely affected during muscular exercise but not during passive exposure to a hot environment [3]. Reductions to both working memory capacity and the analysis and retention of visual information have been observed when core temperature was actively increased to 38.5°C [4]. Based on these contrasting results literature reviews have concluded that cognitive disturbances resulting from hyperthermia are task complexity dependent [9, 12-14].

Exercise could be a possible confounding variable in these previous studies. To date, the effect of passive hot exposure on cognitive function has been poorly investigated. Simmons et al. [15] observed faster reaction times but a loss of accuracy as skin and core temperatures were passively increased. However, a confounding factor in that study could have been an order effect with participants being evaluated in thermoneutral conditions before being retested in a hot environment [15]. Therefore, using a counter-balanced design, the primary objective of this study was to investigate the effect of passive hyperthermia on cognitive function. Based on previous observations following an actively induced hyperthermia [4], we hypothesised that working memory task performance would be altered during passive hyperthermia. However, given the importance of task complexity [9, 12-14], attention tests would not be adversely affected by passive hyperthermia.

From a practical perspective, the higher accident rates and risky behaviour observed in hot environments [16] have been related to increased thermal stress [14]. Errors in judgement and decision making can have severe consequences on the health and safety of individuals working in a hot environment [17]. However, localised cooling of the head has been shown to improve physiological stress [18] and perceived thermal comfort [15, 19]. Although there is a potential practical application to this procedure, the literature remains equivocal over its effectiveness on preserving cognitive function [1, 20]. In addition, the previous studies investigating the effect of head cooling in a hot environment have not included specific memory tasks [1, 20]. Therefore, the second aim of this study was to investigate what specific cognitive functions could be preserved in hyperthermia by applying cold packs to the head.

## Methodology

### Subjects

Sixteen subjects (11 men and 5 women, 31 ± 1 year, 73 ± 3 kg and 175 ± 3 cm for age, weight and height, respectively) gave informed consent to participate in this study which was approved by the Institutional Ethics Committee. The study was designed in accordance with the 1964 Helsinki Declaration.

### Procedure

One week before commencing the experimental trials, subjects completed a familiarisation session comprising the full battery of cognitive tests in an environmental chamber set at 20° C and 40% rH. Using a counter-balance design each subject then completed three experimental trials under each of the following conditions; a control session (CON, 20° C and 40% rH for temperature and humidity, respectively) and two sessions in a hot environment (50°C and 50% rH for temperature and humidity, respectively) with (HHC) or without (HOT) keeping the head cool. Three cool packs (Nexcare, 3M, St Paul, MN, 25 cm × 10 cm, 300 g) frozen at -14°C were applied to the head along the frontal axis and one to the neck across a sagittal axis before entering the environmental room for the HHC session. The cool packs were applied with a protective layer to the skin, maintained in position by a muff net and renewed at approximately 20 min intervals during the entire HHC session (i.e., walk, rest and testing). All sessions were conducted in an environmental chamber (Tescor, Warminster, PA, USA), which controlled temperature and humidity. Sessions were separated by 1 week and performed at the same time of day with subjects dressed in shorts and t-shirt with *ad libitum* water hydration. Body weight was recorded with an electronic scale (precision 0.1 kg) before entering and after exiting the environmental chamber. Each session (CON, HOT and HHC) started with 10 to 15 min walking at 3 to 5km h^−1^ (depending on each subject's fitness level) on a motorised treadmill (T170, Cosmed, Rome, Italy) to initiate heat production during the following resting period without fatiguing the participants. Subjects then rested in a seated position for 45 min before commencing the cognitive assessments.

### Cognitive testing

**Figure 2:**
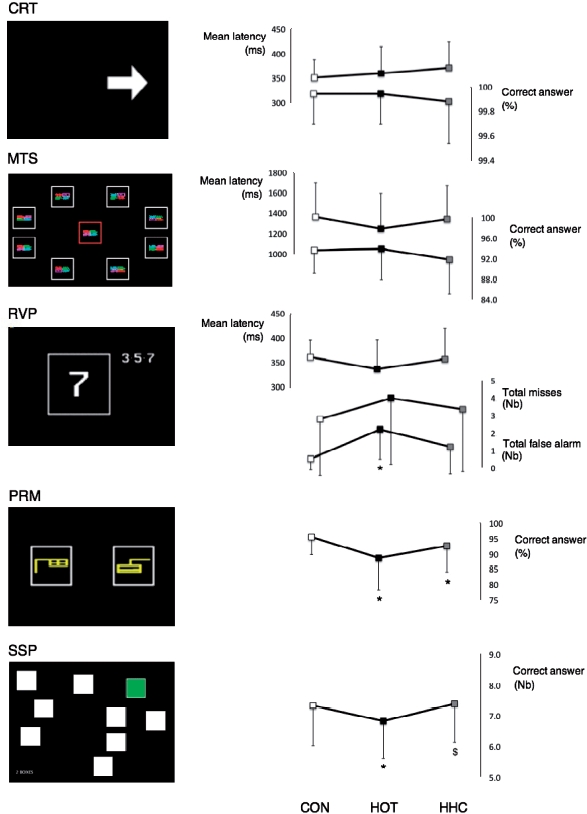
Test screens (left panel) and results (right panel) of cognitive assessments in control (CON, white marks), hot (HOT, black marks) and hot head cool (HHC, grey marks) conditions. CRT, choice reaction time; MTS, match to sample visual search; RVP, rapid visual processing; PRM, pattern recognition memory; SSP, spatial span. *significant impairment as compared to CON (*P*< 0.05); $significant improvement as compared to HOT (*P*< 0.05).

Wearing noise-reducing headphones, subjects performed five different cognitive tests from the Cambridge battery (CANTABeclipse, Cambridge Cognition, Cambridge, UK) inside the environmental chamber. The order of the different cognitive tests was randomised to reduce the potential effect of rising body temperatures throughout the 33 min battery. Each session was performed under constant light (2121x), wind speed (0.5–0.6ms^−1^) and noise conditions. The test battery was performed in a seated position with a 33.8 cm touch screen placed on a desk in front of the subjects. Tests evaluating both simple (attention tests, i.e. less than one second response time [21]) and complex (memory tests [22]) cognitive functions were selected. Figure 2 gives screen examples of each of the tests conducted. The CANTAB battery has been previously validated [23, 24] and used in more than 600 peer-reviewed articles.

### Attention tests

The Match to Sample (MTS) visual search, duration 9 min, tests the ability to match images to a model. This test is used to reflect the functioning of the frontal lobe [25]. An abstract pattern composed of four coloured elements was presented in the centre of the screen. After ∼2 s delay, two, four or eight similar images appeared in boxes around the The subjects were required to recognise the correct image as fast as possible. After a practice at each level of difficulty, the subject randomly performed 6 trials at each difficulty level. The outcome measures were the global percentage of correct answers (accuracy) and the mean correct reaction time (reaction time).

During the choice reaction time test (CRT), duration 7min, subjects had to press the left hand button on a press pad if the stimulus (an arrow) was pointed to the left hand side of the screen, and the right hand button if the stimulus was pointed to the right. Stimuli were displayed with a randomised delay and subjects were asked to respond as quickly as possible. One practice stage with 24 trials and two assessment stages, of 50 trials each were performed. The outcome measures were the mean latency (*n* = 100, reaction time) and the percentage of correct responses (accuracy).

The rapid visual information processing test (RVP), duration 7min, measures sustained visual attention to reflect the functioning of the fronto-parietal regions of the brain [26]. Numbers from 2 to 9 were presented at a rate of 100 digits per min in the centre of the screen in a random order. The subjects had to detect target sequences (2-4-6, 3-5-7 and 4-6-8) and register these on a press pad. The test was in two parts: a two-minute practice stage that was not scored, and a three-minute test stage. For scoring purposes, the number of responses that occurred within 1800 ms of the final digit presented for each of the target sequences was calculated. The outcome measures were the number of missed sequences (accuracy) and the mean latency (reaction time). The number of false alarms was also recorded (impulsivity).

### Memory tests

The spatial span (SSP, duration 5 min) test measures working memory capacity and is sensitive to disturbances in frontal lobe function [27, 28]. Subjects had to remember a series of squares illuminated in a random order on the screen. The number of boxes in the sequence increased from a starting level of two at the start of the test to a final level of nine. There were three possible attempts at each level; however, as soon as the subject successfully passed a sequence at each level they progressed to the next level. If all three sequences were unsuccessfully completed, the test was terminated. The order and colour used was changed from sequence to sequence to minimise interference. The outcome measure was the highest number of squares recalled in the correct order.

Pattern recognition memory (PRM, duration 5 min) measures visual memory and is sensitive to temporal [25] and medial temporal [29] lobe dysfunctions. Subjects were presented with a series of 12 visual patterns, one at a time every 3 s in the centre of the screen. These patterns are designed so they cannot easily be given verbal labels. In the recognition phase, subjects were required to choose between a pattern they have already seen and a novel pattern. In test phase the patterns were presented in the reverse order to the recognition phase. The outcomes measure was the percentage of correct responses of two series of 12 different patterns. To reduce any possible learning effect, three different groups of patterns were randomly assigned such that subjects never saw the same group twice throughout their three testing sessions (i.e. CON, HOT and HHC).

### Temperature recordings

A wireless Mini Mitter Jonah™ ingestible thermometer pill was swallowed at least 5 h before each trial to measure T_core_. Forehead temperature (T_head_) was monitored with a XTP wireless dermal adhesive temperature patch. Both sensors sent data (precision 0.01°C) by telemetry every 60 s to the VitalSense® recording system (Mini Mitter, Respironics, Herrsching, Germany). Tympanic temperature (T_tymp_) was recorded with an infrared thermometer (MP7 Qiuick, Medel, S. Polo di Torrile, Italy). A timestamp for each temperature was recorded on four separate occasions in each condition; pre-entry into the chamber, at the end of the walk, and at the start and end of the cognitive tests.

### Statistical analysis

All statistical analyses were performed using Predictive Analytics Software (PASW Version 18.0). A two-way within subject analysis of variance (ANOVA) for repeated measures (Time versus Condition) was used to assess for main effects of Condition (CON or HOT or HHC) and Time (pre-entry, end of walk, start of cognitive test, and end of cognitive test) on changes in temperature (T_core_, T_head_, T_tymp_) at the different points. In addition, oneway ANOVA for repeated measures with single factor Condition (CON, HOT or HHC) was used to assess changes in cognitive functions. In addition, two-way within subject analysis of variance (ANOVA) for repeated measures (Time versus Condition) was used to assess for main effects of Condition (CON or HOT or HHC) and Time (pre-entry and end of cognitive test) on changes in body weight during sessions. Prior to ANOVA tests, data was screened for normality, homogeneity of variance using the F-Max test and sphericity using Mauchly's test. In cases of violation, Greenhouse-Geisser epsilon corrections were used to adjust degrees of freedom. Where a significant interaction was found, post hoc pairwise comparisons were performed with Bonferroni tests. Data is presented as mean ± SD and statistical significance was accepted at *P* < 0.05.

## Results

**Figure 1:**
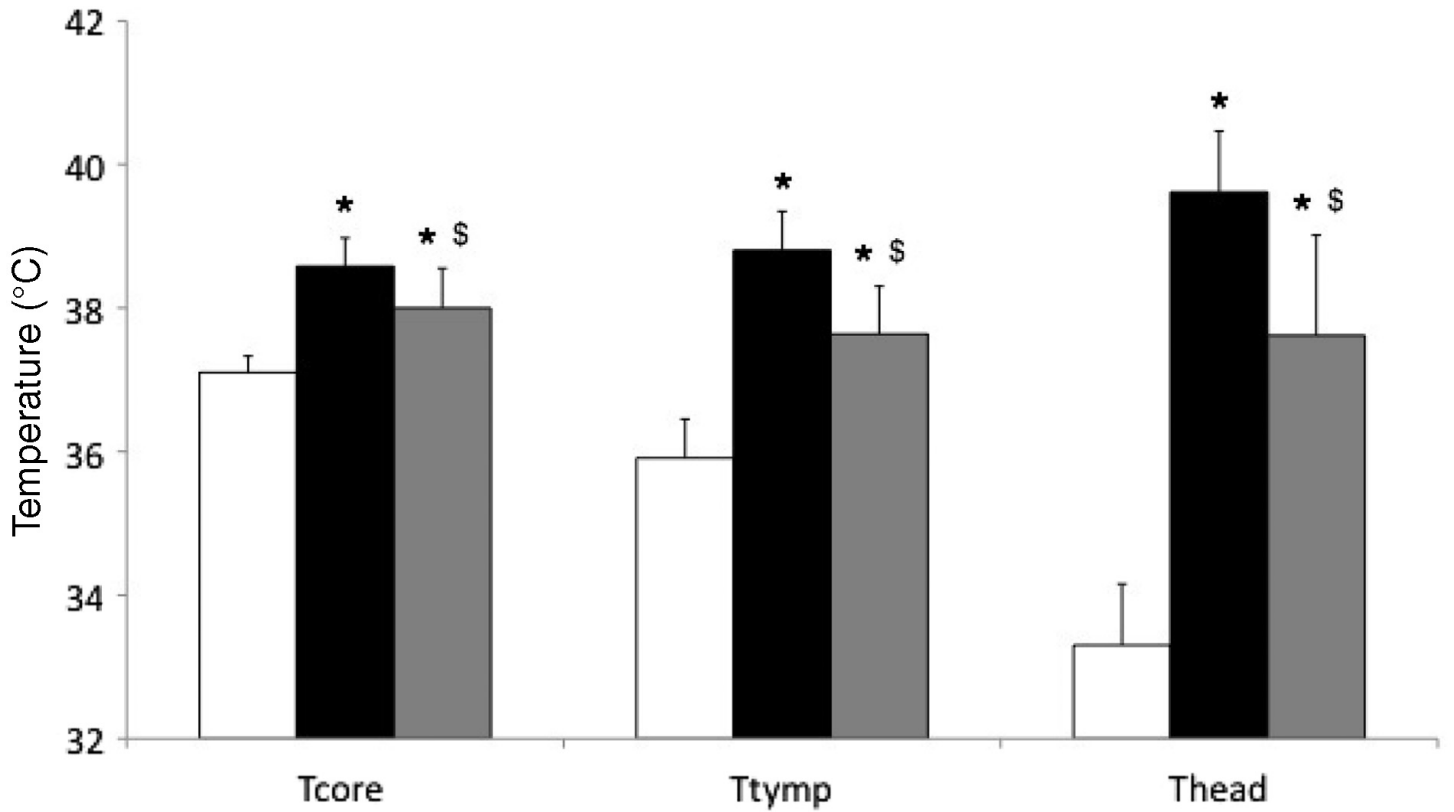
Average temperatures in control (CON, white bars), hot (HOT, black bars) and hot head cool (HHC, grey bars) conditions during cognitive testing. T_core_, intestinal temperature; T_tymp_, tympanic temperature; T_head_, skin temperature on forehead. *Significant higher values than in CON (*P*< 0.001); $significant lower values than in HOT (*P*< 0.001).

There were significant global effects for time (F_1.8,20.1_ = 51.0, *P* < 0.001), condition (F_1.4,15.1_ = 20.7, *P* < 0.001) and interaction (F_2.4,26.3_ = 23.9, *P* < 0.001) upon T_core_. There were significant effects for time (F_3,30_>36.3, *P* < 0.001), condition (F_2,20_>57.8, *P*< 0.001) upon T_head_ and T_tymp_. Significant time and condition interactions were found for T_head_ (F_6,60_ = 25.7, *P* < 0.001) and T_tymp_ (F_2.9,29.5_= 16.5, *P* < 0.001). Post hoc analysis revealed that before entering the chamber there were no differences between conditions in T_core_ (mean 37.1° ± 0.2°C), T_head_ (mean 32.7° ± 0.5°C) or T_tymp_ (mean 36.0° ± 0.5°C). Only in the HHC (0.36°C, 95%CI (0.11, 0.61) *P* = 0.003) did the walk completed upon entry into the environmental chamber increase T_core_. There was no change in T_core_ in CON from the end of the walk to the start of the cognitive testing, however, in HOT (1.1 °C, 95%CI (0.64, 1.56) *P* < 0.001) and HHC (0.65°C, 95%CI (0.14, 1.17) *P*=0.01) mean T_core_ significantly increased during the resting period. Temperature plateau thereafter and there was no change in T_core_, T_tymp_ and T_head_ from the beginning to the end of the cognitive trials in any condition. Consequently, mean temperatures during the cognitive sessions were used for between condition comparisons and these are shown in Figure 1. Mean T_core_ (F_1.3, 18.7_ = 45.4, *P* < 0.001), T_tymp_p (F_2,26_ = 84.1, *P* < 0.001) and T_head_ (F_2,28_= 155.7, *P* < 0.001) were significantly higher during both the HOT and HHC cognitive trials than during CON (Figure 1). In addition, compared to HOT, cooling the head reduced T_core_, T_tymp_ and T_head_ by 0.6°±0.2°C (*P*= 0.004), 1.1°±0.2°C (*P*= 0.001) and 1.9°±0.4°C (*P*= 0.002), respectively.

Body weight remained unchanged between the different conditions (F_2,30_ = 0.08, *P* = 0.923) while pre- and post-body weight did not change (F_1,15_ = 2.07, *P*=0.17) in any condition, such that body weight changes did not exceed 0.5%.

### Cognitive testing

Results of the cognitive testing are displayed in Figure 2.

### MTS, CRT and RVP

Our data showed no differences in the reaction time and the accuracy of the attention tasks between CON, HOT and HHC (MTS: F_2,30_=1.24 and F_2,30_=1.02; CRT: F_2,30_=1.57 and F_2,30_ = 0.32;

RVP: F_2,30_ = 2.45 and F_2,30_=1.34; for reaction time and accuracy, respectively).

However, impulsivity during the RVP task displayed a session effect (F_2,30_ = 6.61, *P* < 0.01) with a significantly higher number of false alarms in HOT than in CON (*P*<0.01). Head cooling reduced the number of false alarms compared to HOT but did not reach significance.

### SSP

The longest sequence correctly recalled during the SSP was condition dependent (F_2,30_ = 4.16, *P* = 0.025) and significantly longer in both CON (0.5, 95%CI (0.1,0.9) *P* = 0.027) and HHC (0.6, 95%CI (0.2,1.0) *P* = 0.007) than HOT. There was no difference in SSP performance recorded in HHC as compared to CON (0.1, 95%CI (-0.5,0.6) *P* = 0.806).

### PRM

The percentage of correct answers was condition dependent (F_1.4,20.7_ = 4.21, *P* = 0.042) and was significantly lower in HOT than in CON (6.8, 95%CI (1.3,12.3) *P* = 0.023). There was no significant difference between HOT and HHC (-3.9, 95%CI (-9.2,1.4) *P* = 0.023); however, there was no difference between HHC and CON (2.9, 95%CI (0,5.8) *P* = 0.048).

## Discussion

The main findings of this experiment were that impairments in cognitive function with passive hyperthermia and their preservation via head cooling are dependent upon the type of task performed.

### Attention tests

*Reaction time:* Improved reaction times have previously been observed in hot environments [15, 30]. However, the current results show that when T_core_ increased to 38.7°C the beneficial effect of heat exposure on reaction time disappears. The reaction times recorded in this experiment (i.e. MTS, CRT and RVP) were dependent on both the time needed to analyse the information and the latency of the transmission of the motor drive to the finger pressing on the pad/screen. As nerve conduction velocity increases in a hot environment, there is a decrease in the transmission latency of an action potential (physiological data of the same experiment, Racinais et al. [31]). Therefore, the absence of a modification in reaction time suggests an increase in motor drive velocity can compensate for the altered treatment of information processing. Prolonged heat stress has been shown to have detrimental effects on reaction time [14]. Neave et al. [32] observed that wearing a helmet during exercise, which potentially induced cerebral hyperthermia, altered attention, vigilance and slowed reaction times. However, reaction times were not altered in the current experiment with a passive heat exposure that induced higher body temperatures than in Neave et al. [32], which suggests that exercise, not hyperthermia per se, was responsible for the slower reaction times in the Neave et al. [32] experiment.

*Accuracy:* Studies observing an improvement in reaction time have also reported an associated loss of accuracy [15, 30]. In the current experiment, the absence of a significant difference in reaction times between conditions was not associated with an increase in the number of errors in both the MTS and CRT.

*Impulsivity:* In contrast to the CRT and MTS tests, the RVP task requires sustaining attention over a continuous period and working memory to recall the target and the previous digits [26]. In this more demanding task the number of false alarms increased with hyperthermia. This result supports previous research that has suggested that tasks demanding lower attention are less vulnerable to the effects of hyperthermia than those tasks requiring more attention [14].

Even in thermo-neutral conditions, maintaining attentional resources for prolonged periods of time can create a fatiguing mental load [33-35] that is related to deterioration in cognitive performance [36]. Previous studies have shown in thermoneutral conditions that decrements in sustained attention appear within 20 to 35 min, depending on the task [37, 38]. However, the current study revealed that a hot environment substantially reduced this time limit such that participants showed an increased level of impulsivity during a 7-min task.

### Memory tests

Impairments of complex cognitive functions, such as memory, have been observed after dehydration following both active [5] and passive hyperthermia [5, 39]. While reductions of more than 2% body weight have failed to influence simple cognitive functions [8, 40], it is generally accepted that a heat-induced dehydration inducing a body weight loss greater than 2% adversely affects more complex cognitive performance [32, 41]. In the current experiment, body weight changes did not exceed 0.5%, suggesting that fluid replacement was adequate and unlikely to have negatively influenced cognitive performance [15, 32]. Consequently, the decrements observed in working memory tasks in the current study can be related to hyperthermia rather than dehydration.

The present study observed significant decreases in SSP and PRM performance with passive heat exposure. This could be explained by a physiological alteration of the brain, related to the heating of the cortical neurons. This hypothesis is supported by studies that have observed a change in the electrical activity of the brain (i.e. encephalogram (EEG) activity and sensory evoked potentials) when exercising in hot environments [4, 42, 43] and with passive hyperthermia in primates [44]. In humans there is a dearth of information during passive hyperthermia and only exercise models have been used to investigate the EEG activity of exercising humans. Nielsen et al. [43] observed a reduction in high frequency b band inducing a rise in the a/b index when T_core_ increased during exercise. Although exercise could be seen as a confounding variable, the larger increases in a/b index were recorded in the hot condition where the final T_core_ was significantly higher than the control condition despite a similar exercise load. This suggests that the alterations in the electrical activity in the brain's frontal area resulted from hyperthermia-associated fatigue [43]. It has been shown that hyperthermia can induce EEG frequency changes obtained over the prefrontal [45], frontal [43] and occipito-parietal regions [4] of the brain. These areas have been shown to influence the performance to the cognitive tests utilised in the current study. In fact, visual working memory (PRM) is sensitive to temporal [25] and medial temporal [29] lobe dysfunctions, while the SSP, a computerised version of the Corsi block task, is sensitive to disturbances in frontal lobe function [27, 28].

### Head cooling

The second aim of this study was to investigate whether specific cognitive functions could be preserved through the regular application of cold packs to the head during heat exposure. Previous studies have reported the protective benefits of head cooling on both the physiological response [1, 30] and cognitive task performance in stressful environments [46]. During the attention tests in the current study only the number of false alarms (RVP) was altered by hyperthermia. Applying cool packs to the head partially reversed this alteration (Figure 2). Previously, Simmons et al. [15] did not observe any beneficial effect of head cooling on a similar RVP task as performed in the current study. This could be due to fact that the forehead, which has the most beneficial effect on reducing thermoregulatory responses to a hot environment [18], was not directly cooled in the Simmons et al. study. Consequently, the ∼ 2°C reduction in T_head_ recorded in the current study was higher than in Simmons et al. [15] (1.3°C). Another other possible explanation for the contrasting results is that the RVP task used in the current study is sensitive to frontal lobe dysfunction [26], and consequently this could explain the beneficial effect of head cooling on this task.

Although both memory task performances decreased in HOT, the regular application of cold packs on the head only prevented the detrimental effect of hyperthermia on short-term memory capacity (SSP, Figure 2). It was ineffective in protecting visual memory (PRM) performance. The contrasting effect of head cooling during hyperthermia on SSP and PRM could be explained by these tests reflecting the cognitive performance of two different areas of the brain: frontal [27, 28] and temporal [25, 29], respectively. As with the RVP task, the reduction in T_head_ values observed following the application of cool packs provides additional support for the protective effect of direct cooling to the frontal area of the brain.

The temperatures of both the frontal and temporal lobes are correlated to T_core_ [47]. However, each brain area has its own homeostatic temperature [48], suggesting that at the same T_core_ the thermal load between the different brain areas could be different. The varying thermal load during heat exposure becomes more important, as when at rest the cooler brain regions exhibit significantly lower neu-ronal activity than the warmer regions [49].

Hocking et al. [4] hypothesised that task performance would deteriorate when the total cognitive resources are insufficient for both the task and the thermal stress. Following this model, reducing the thermal stress to a particular brain area should reduce its specific load and, consequently, recover its capacity to execute the corresponding cognitive functions. The current data confirm that the beneficial effect of head cooling is task specific. This may be related to the fact that each brain area has its own homeostatic temperature, with a dorso-ventral temperature gradient being exhibited in humans [48]. However, the responsible underlying mechanisms are still unknown, and further investigations using EEG and brain mapping are recommended.

### The overload paradigm

A previous study revealed interference between two concurrent tasks requiring activation of the same part of the neural cortex [50]. Subsequently, interference has been observed between two cognitive tasks [51-55], two motor tasks [56-58], the combination of cognitive and motor tasks in a neutral environment [36, 59-62], and during exercise-induced fatigue in a hot environment [4]. The current data add to these findings, and suggest that passive heating can also be considered as a load that interferes with cognitive processes.

Steady-state probe topography has shown that steady-state visual-evoked potentials increase in amplitude and decrease in latency in the frontal lobe for working memory tasks, and in occipito-parietal regions for vigilance tasks when experiencing thermal strain [4]. These results suggest that the brain uses greater neural resources in order to maintain the same performance despite the thermal strain, until the resources are overloaded [4], which may explain why only complex cognitive tasks were impaired by passive hyperthermia in the current study. This suggests that cognitive task performance deteriorates when the total resources are insufficient for both the task and the thermal stress imposed on a specific brain area due to humans having limited cognitive capacity with external stimuli competing to access the limited global workspace [63, 64].

## Conclusion

Impairments in cognitive function with passive hyperthermia are task specific. Hyperthermia impairs working memory but does not alter in simple attentional processes. The beneficial effect of head cooling is also task dependent and appears to be more efficient with cognitive functions primarily involving the frontal area of the brain.
